# High frequency of the erythroid silent Duffy antigen genotype and lack of *Plasmodium vivax* infections in Haiti

**DOI:** 10.1186/1475-2875-12-30

**Published:** 2013-01-24

**Authors:** Thomas A Weppelmann, Tamar E Carter, Zhongsheng Chen, Michael E von Fricken, Yves S Victor, Alexander Existe, Bernard A Okech

**Affiliations:** 1 Department of Environmental and Global Health, University of Florida, PO Box 100188, Gainesville, FL 32610, USA; 2 Emerging Pathogens Institute, University of Florida, 2055 Mowry Rd, P.O. Box 100009, Gainesville, FL 32610, USA; 3 Genetics Institute, University of Florida, 2033 Mowry Rd, PO Box 103610, Gainesville, FL 32610, USA; 4 Department of Anthropology, University of Florida, 1112 Turlington Hall, PO Box 117305, Gainesville, FL 32611, USA; 5 Department of Epidemiology, College of Public Health and Health Professions, University of Florida, PO Box 100231, Gainesville, FL 32611, USA; 6 Blanchard Clinic, Family Health Ministries Haiti, Terre Noire, Port au Prince, Haiti; 7 National Public Health Laboratory, Ministry of Public Health and Population (MSPP), Delmas 33, Port au Prince, Haiti

**Keywords:** *Plasmodium vivax*, Malaria, Hispaniola, Haiti, Duffy antigen receptor for chemokines (*DARC*), Duffy negative, Genetic susceptibility

## Abstract

**Background:**

Malaria is a significant public health concern in Haiti where approximately 30,000 cases are reported annually with CDC estimates as high as 200,000. Malaria infections in Haiti are caused almost exclusively by *Plasmodium falciparum*, while a small number of *Plasmodium malariae* and an even smaller number of putative *Plasmodium vivax* infections have been reported. The lack of confirmed *P. vivax* infections in Haiti could be due to the genetic background of native Haitians. Having descended from West African populations, many Haitians could be Duffy negative due to a single nucleotide polymorphism from thymine to cytosine in the GATA box of the promoter region of the Duffy antigen receptor for chemokines (*DARC*) gene. This mutation, encoded by the *FY*^*ES*^ allele, eliminates the expression of the Duffy antigen on erythrocytes, which reduces invasion by *P. vivax*. This study investigated the frequency of the *FY*^*ES*^ allele and *P. vivax* infections in malaria patients with the goal of uncovering factors for the lack of *P. vivax* infections reported in Haiti.

**Methods:**

DNA was extracted from dried blood spots collected from malaria patients at four clinic locations in Haiti. The samples were analysed by polymerase chain reaction (PCR) for the presence of the *P. vivax* small subunit ribosomal RNA gene. PCR, sequencing, and restriction enzyme digestion were used to detect the presence of the *FY*^*ES*^ allele. Matched samples were examined for both presence of *P. vivax* and the *FY*^*ES*^ allele.

**Results:**

No cases of *P. vivax* were detected in any of the samples (0/136). Of all samples tested for the *FY*^*ES*^ allele, 99.4% had the *FY*^*ES*^ allele (163/164). Of the matched samples, 99% had the *FY*^*ES*^ allele (98/99).

**Conclusions:**

In this preliminary study, no cases of *P. vivax* were confirmed by PCR and 99% of the malaria patients tested carried the *FY*^*ES*^ allele. The high frequency of the *FY*^*ES*^ allele that silences erythroid expression of the Duffy antigen offers a biologically plausible explanation for the lack of *P. vivax* infections observed. These results provide insights on the host susceptibility for *P. vivax* infections that has never before been investigated in Haiti.

## Background

Malaria transmission in Haiti is a serious public health concern where approximately 30,000 cases are reported each year with Centers for Disease Control and Prevention (CDC) estimates as high as 200,000 [1]. Over 99% of malaria infections in Haiti are caused by *Plasmodium falciparum*, with less than 1% of cases reported as *Plasmodium malariae* infections [2-4]. There are even fewer reports of *Plasmodium vivax* in Haiti, all of which were only detected by microscopy and none confirmed by polymerase chain reaction (PCR) based methodologies [2,5,6]. Since *P. vivax* is responsible for 74% of all cases of malaria in Central and South America and up to 94% of cases in Mexico and Central America, the lack of *P. vivax* infections in Haiti merits further investigation [1,5,7]. Besides geographical isolation, host genetic factors could be another reason for the lack of *P. vivax* infections in Haiti. A majority of Haitians are descendants of West African populations, who have a high frequency of individuals that do not express the Duffy antigen receptor for chemokines (*DARC*) on their erythrocytes [8,9]. The absence of the Duffy antigen on erythrocytes has been shown to reduce or completely prevent *P. vivax* infections [10-14].

The Duffy antigen is a trans-membrane glycoprotein located on red blood cells that contains a N-terminal domain used by *P. vivax* merozoites to invade and infect erythrocytes [15,16]. There are two co-dominant alleles *FY*^*A*^ and *FY*^*B*^ that differ by a single nucleotide at amino acid codon 42, where a change from glycine to aspartic acid results in the expression of the Fy^a^ or Fy^b^ antigen, respectively [17]. In many African and African-descendent populations the expression of the Duffy antigen is silenced as a result of a single nucleotide polymorphism (SNP) in the GATA box sequence in the *DARC* gene promoter region in which a cytosine (C) replaces thymine (T) 46 nucleotides before the erythroid cap site [18-20]. This erythroid silent (*ES*) genotype, encoded by the *FY*^*ES*^ allele, is responsible for the Duffy negative phenotype expressed by homozygotes that can reach a population frequency of 100% in native West Africans [9,21].

Neither the reason why *P. vivax* has been so rarely observed in Haitians, nor the prevalence of the erythroid silent Duffy antigen genotype has been investigated in detail. Therefore, this study investigated the frequency of the *FY*^*ES*^ allele and *P. vivax* infections in malaria patients. The information obtained from this study will help clarify the status of *P. vivax* infections in Haiti.

## Methods

### Data collection

**Figure 1:**
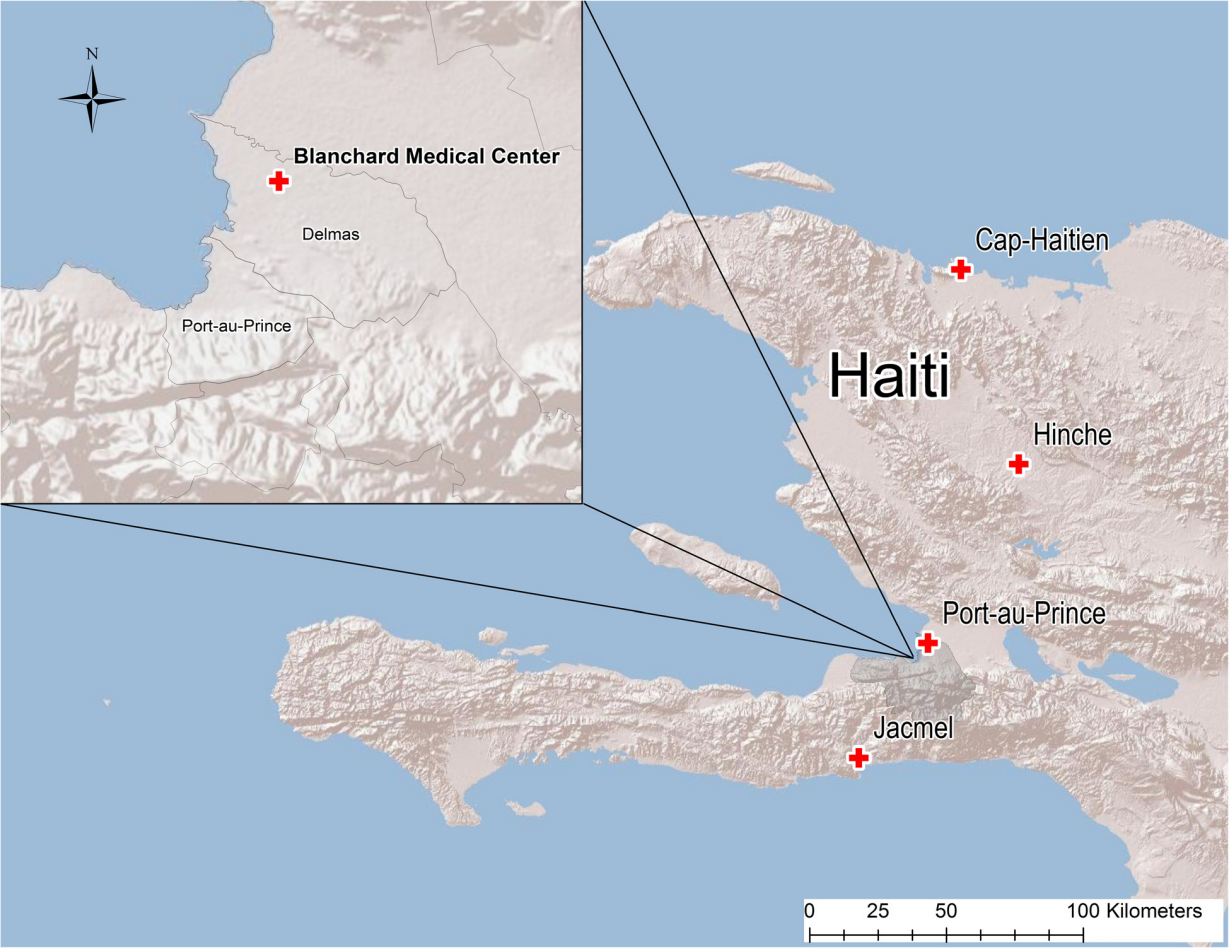
Map of Haiti with clinical origin of dried blood spots.

Dried blood samples (DBS) were collected by finger prick and blotted on filter paper (FTA cards, Whatman©) in Haiti from May 2010 to August 2012. The samples originated from several clinics including the Blanchard clinic in Port-au-Prince (n = 164), Hinche (n = 7), Cap-Haitien (n = 7), and Jacmel (n = 6) (Figure 1). Samples from patients at the Blanchard clinic included symptomatic individuals as well as those confirmed by rapid test and/or microscopy to have *P. falciparum* or mixed infections (*P. falciparum* and *P. malariae* or *P. vivax*). Samples from patients at the other three locations were confirmed by microscopy alone. The DBS were transported to the Emerging Pathogens Institute at the University of Florida and archived in dry storage before further genetic analyses as described below.

### DNA extraction

A methanol extraction procedure was used to extract the DNA from the DBS [22]. Three millimetre discs were punched from the DBS, placed inside a PCR tube, and 100 μl of absolute methanol was added. The samples were incubated at 25 degrees Celsius (°C) for 15 minutes, the methanol was removed, and the samples were allowed to dry overnight. Once dry, 65 μl of nuclease free water was added and heated to 95°C for 30 minutes. The samples were centrifuged and then stored at 4°C for immediate use or stored at – 20°C for future use.

### Detection of *P. vivax* by polymerase chain reaction

A species-specific PCR protocol was used to detect the presence of *P. vivax* in the samples. PCR primers were targeted to detect the *P. vivax* small subunit ribosomal RNA gene and were able to detect low levels of parasites [23]. The forward primer and reverse primer sequences used were 5^′^ GCAACGCTTCTAGCTTAATC 3^′^ and 5^′^ACAAGGACTTCCAAGCCGAAGC 3^′^, respectively. The PCR temperature protocol was as follows: 94°C for 5 minutes; 94°C for 30 seconds, 58°C for 30 seconds, and 72°C for 60 seconds for 40 cycles; and 72°C for 10 minutes. The expected size of the amplicon was 131 base pairs (bps). All PCR runs included a *P. vivax* positive control obtained from the malaria reagents and resources center (MR4).

### PCR and sequencing of the *DARC* Gene

A PCR protocol was used to amplify a segment of the *DARC* gene that was 329 bps and included the –46 T/C SNP [14]. The forward primer and reverse primer sequences used were 5^′^CAGGAAGACCCAAGGCCAG 3^′^ and 5^′^CCATGGCACCGTTTGGTTCAGG 3^′^ respectively. The PCR temperature protocol was as follows: 94°C for 5 min; 94°C for 20 seconds, 62°C for 15 seconds, and 72°C for 20 seconds for 35 cycles; and 72°C for 10 minutes.

Ten of the samples from the malaria patients along with a blood sample from one of the authors of European ancestry were sequenced for the GATA box mutation with the forward and reverse primers described above to identify –46 T and –46C controls for use in the restriction enzyme digestion of patient samples. DNA was sequenced by the Interdisciplinary Center for Biotechnology Research at the University of Florida using Sanger sequencing. Sequence analysis was accomplished by the examination of the chromatograms with 4Peaks (Mekentosj, Amsterdam, NL) software followed by alignments using Clustal *X*2 [24].

### Restriction fragment length polymorphism of DARC

Restriction fragment length polymorphism (RFLP) analysis was used to confirm the presence or absence of the −46 T/C SNP [14]. The PCR products were digested overnight with the restriction endonuclease *Sty* I (New England Biolabs, Ipswich, MA) according to the manufacturer’s specifications. After heat inactivation of the enzyme for 10 minutes at 65°C, the fragments were stained with ethidium bromide and resolved by gel electrophoresis on a 4% agarose gel for 2.5 hours at 140 volts.

## Results

The results from the GATA box mutation analysis and the detection of *P. vivax* from the DBS are shown below (Table 1). No cases of P. *vivax* were detected by PCR in 136 patient samples tested from the Blanchard clinic. Of the samples tested for the *FY*^*ES*^ allele from the same clinic, 99.4% (163/164) had the *FY*^*ES*^ allele. Of the matched samples (n =99) tested for both the presence of P. *vivax* and the *FY*^*ES*^ allele, no *P. vivax* infections were found and 99% (98/99) had the *FY*^*ES*^ allele. The remaining samples from the other sites were not tested (NT) for *P. vivax*, however 100% (20/20) were positive for the *FY*^*ES*^ allele.

**Table 1:** **Prevalence of GATA box mutations and *P. vivax *Infections**

**Site of sample**	**(*FY***^***ES***^**) allele presence**			***P. vivax *presence**	
**Collection**	**No. tested**	**No. positive**	**% Positive**	**No. tested**	**No. positive**
Blanchard Clinic	164	163	99.4	136	0
Hinche	7	7	100	0	NT*
Cap-Haitien	7	7	100	0	NT*
Jacmel	6	6	100	0	NT*
Total	184	99.5%	99.5%	136	0

*NT represents samples not tested for the presence of *P. vivax.*

## Discussion

This preliminary study is the first in Haiti to focus on the prevalence of the *FY*^*ES*^ allele and *P*. *vivax* infections in malaria patients. No *P. vivax* infections were found and 99% of the malaria patients had the *FY*^*ES*^ allele. The frequency of the *FY*^*ES*^ allele observed in this study could explain the different rates of *P. vivax* Haiti as compared to Central and South America, where the majority of infections are *P. vivax* and the population frequency of the *FY*^*ES*^ allele is rare [17,25]. The results from this study are similar to other studies conducted in West African populations, who have between 95-100% prevalence of the *FY*^*ES*^ allele and almost complete absence of *P. vivax* infections. In a related study in Gambia (n = 1,176) where the population had complete penetrance of the Duffy negative phenotype, of the samples positive for the presence of malaria parasites, 94.8% contained *P. falciparum*, 3.7% contained *P. malariae*, 1.5% contained *Plasmodium ovale*, and 0% contained *P. vivax*[21]. In another study from nine countries in West and Central Africa (n = 2,588) where the Duffy negative genotype is estimated to be above 95%, PCR analysis of blood samples indicated that of samples positive for malaria parasites, only a single case of *P*. *vivax* was found while 98.5% contained *P. falciparum*, 8.5% contained *P. malariae*, and 3.9% contained *P. ovale*[9].

The high frequency of the *FY*^*ES*^ allele found in this study could be attributed to the West African ancestry of Haitians. Historical records dating back to the 1780s indicate that a large proportion of West Africans brought to the New World during the trans-Atlantic slave trade arrived in Haiti [8]. It is estimated that over 75% of the total number of enslaved Africans in Haiti came from West and West Central Africa, which have almost exclusively the *FY*^*ES*^/*FY*^*ES*^ genotype [8,9]. This study found only a single patient without the *FY*^*ES*^ allele that may be due to the small proportion of Haitians having French and/or Spanish ancestry or a foreign aid worker, who have much lower population frequencies of the *FY*^*ES*^ allele [18]. Though the French and the Spanish originally colonized Haiti, in 1804 the French departed and the remaining Spanish sequestered themselves eastward to the Dominican Republic. This massive gene flow from West Africa followed by the establishment of Haiti as an independent country could have contributed to high population frequency of Duffy negative individuals [8].

Although the sample size was relatively small, no *P. vivax* DNA was found in any of the patient samples, which is consistent with previous reports of the lack of *P. vivax* in Haiti [2-4]. It is likely that the high prevalence of the *FY*^*ES*^ allele that causes the Duffy negative phenotype may be responsible for the lack of *P. vivax* transmission in Haiti. The samples in this study were obtained from areas that are predominantly of West African ancestry, which are more representative of the ancestry of native Haitians. However, historical reports have indicated *P. vivax* transmission in communes inhabited by higher proportions of people who are of African/European descent that have lower population frequencies of the Duffy negative phenotype [26]. Since *P. vivax* infections have received little attention, it is unknown if such transmission is still occurring in those areas of Haiti. It is likely that low rates of travelers to Haiti from the surrounding *P. vivax* endemic countries in South America may have slowed any potential introductions of *P. vivax* infections into Haiti. Even if introductions were to occur, it would not be likely that *P. vivax* transmission could be sustained because of the high rates of the *FY*^*ES*^ allele in the population. A more extensive study with a larger sample size from multiple sites in Haiti is desirable to confirm these results.

In conclusion, no *P. vivax* infections were confirmed by PCR and the *FY*^*ES*^ allele that results in silenced expression of the Duffy antigen on erythrocytes was found in 99% of the patients. The high frequency of *FY*^*ES*^ allele could explain the sparse historical evidence of *P. vivax* infections in native Haitians and is attributable to their West African ancestry. Though *P. vivax* malaria is not prevalent and does not represent a significant public health risk in native Haitians, the risk of infection for travellers and foreign aid workers who are Duffy positive remains uncertain.

## Competing interests

The authors declare that they have no competing interests.

## Authors’ contributions

TAW carried out the genetic testing for the *FY*^*ES*^ allele and drafting the manuscript. TC helped conduct the genetic testing for the *P. vivax* and revision of the manuscript. ZC helped conduct the genetic testing for *P. vivax*. MEV was involved in drafting of the manuscript. VSF, AE aided in the sample collection. BAO conceived, designed and coordinated the study, and helped draft the manuscript. All authors read and approved the final manuscript.
